# A higher dietary inflammation index is associated with the incidence of overactive bladder: Evidence from a cross-sectional study (NHANES 2011–2018)

**DOI:** 10.1097/MD.0000000000048407

**Published:** 2026-04-24

**Authors:** Mingchu Jin, Yuzhu Huang, Xintao Lv, Haidong Hao, Maohua Luo

**Affiliations:** a Department of Urology, Renmin Hospital, Hubei University of Medicine, Shiyan, Hubei, PR China.

**Keywords:** cross-sectional, dietary inflammatory index, NHANES, overactive bladder

## Abstract

Current research has demonstrated that the development of overactive bladder (OAB) is related to the inflammatory response within the organism, and that this inflammatory response is closely associated with dietary factors; Accordingly, the purpose of this study was to investigate the connection between OAB and the dietary inflammatory index (DII). This cross-sectional analysis was based on data drawn from the National Health and Nutrition Examination Survey (NHANES) spanning the years 2011 to 2018, which included 21,263 participants (50.6% female, 49.4% male). The mean age of participants was 50.5 years, with the racial/ethnic distribution as follows: 42.9% non-Hispanic White, 21.1% non-Hispanic Black, 13.9% Mexican American, and 22.1% from other racial/ethnic groups. We employed weighted multiple logistic regression to analyze the correlation between DII and OAB, adjusting for potential confounders. This study comprised 21,263 people from the NHANES dataset. Following adjustment for all potential confounding variables, a positive association was observed between DII and the risk of OAB (OR 1.18, 95% CI 1.16–1.21). Smoothed curve fitting indicated a nonlinear correlation between DII and OAB, with an inflection point at 1.92. Stratified analyses demonstrated that this correlation was stable across different population subgroups. This study identified a significant nonlinear association between DII and OAB syndrome, suggesting that DII may serve as a useful indicator for predicting and diagnosing OAB.

## 1. Introduction

Overactive bladder (OAB) is a prevalent condition characterized by urinary urgency, urge incontinence, frequent urination, and nocturia, significantly affecting patients’ quality of life.^[1-3]^ The current diagnosis of OAB is based on patient history, bladder volume assessment, and urinalysis. Standardized questionnaires are essential tools for assessing symptom severity and evaluating their effects on patients’ quality of life.^[4]^ The prevalence of OAB varies across countries and regions, which can be attributed to factors such as ethnicity and lifestyle habits. OAB has the capacity to exert a substantial influence on the quality of life experienced by patients, with the potential to engender impairment to social functioning to varying degrees. Studies have demonstrated that OAB condition is a highly prevalent one within the United States affecting 17% of men and 30% of women,^[5]^ and that the annual healthcare costs associated with treatment are significant, highlighting the importance of early diagnosis and intervention.^[6,7]^

The pathophysiology of OAB is complex and multifactorial, with inflammation emerging as a key contributing factor. Numerous studies have linked various inflammatory markers to the development of OAB.^[8]^ Conclusive evidence has emerged from previous studies, which have demonstrated that various inflammatory markers, such as systemic inflammatory response index,^[9]^ systemic inflammatory index, and systemic inflammatory syndrome index (AISI),^[10]^ are associated with the development of OAB and may assist in its diagnosis and prediction. The correlation between diet and inflammation has also been clearly established, with specific dietary components influencing the levels of inflammatory mediators in the body.^[11]^ Therefore, the dietary inflammatory index (DII) serves as an important tool for assessing how dietary patterns relate to the risk of OAB.

The development of the DII constitutes a substantial advancement in the field of nutrition science, by providing a standardized approach for the evaluation of the inflammatory potential of different dietary substances.^[12]^ The DII encompasses not only micronutrients and macronutrients but also common bioactive constituents such as flavonoids, spices, and teas.^[13]^ Although the correlation between DII and diseases such as hypertension, stroke, obesity, type 2 diabetes, metabolic syndrome.^[14-17]^ Despite these findings, the relationship between DII and OAB has not been thoroughly investigated. This study aims to fill this gap by exploring the association between DII and OAB using data from the National Health and Nutrition Examination Survey (NHANES).

## 2. Materials and methods

### 2.1. Study population

**Figure 1.**
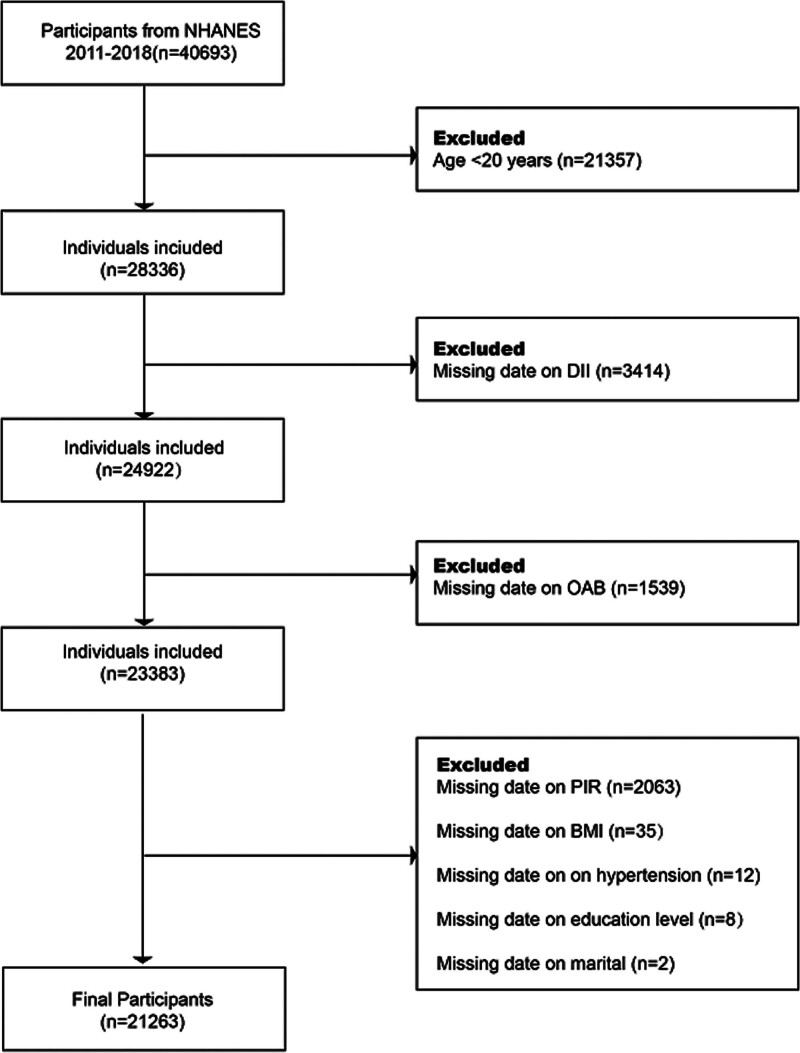
Flowchart of participant selection from NHANES 2011–2018. BMI = body mass index, DII = dietary inflammatory index, NHANES = National Health and Nutrition Examination Survey, OAB = overactive bladder, PIR = poverty–income ratio.

The NHANES is a biennial, cross-sectional survey conducted by the Centers for Disease Control and Prevention (CDC) to evaluate the health and nutritional status of the US population. This flowchart illustrates the participant selection process for the study. Data from the NHANES 2011–2018 were initially collected from 49,693 participants. After excluding individuals based on the following criteria: age under 20 years (n = 21,357), missing OAB data (n = 3414), missing dietary data (n = 1539), and missing relevant covariates (n = 2120), a final sample of 21,263 participants was included in the analysis. This diagram depicts the number of participants at each exclusion step (Fig. 1). The NHANES protocols were approved by the National Center for Health Statistics Research Ethics Review Board, and informed consent was obtained from all participants.^[18]^ All data utilized in this study were de-identified and publicly accessible via the following link: (https://www.cdc.gov/nchs/nhanes/?CDC_AAref_Val).

### 2.2. Assessment of dietary inflammatory index (DII)

The DII is an assessment tool designed to quantify the inflammatory potential of the diet, consisting of 45 food parameters. The dietary data used to calculate the DII were collected using a 24-hour dietary recall interview. This data was accessed from the NHANES Food File, which includes nutrient intake and food group data from each participant’s reported food consumption. A specific DII score was assigned to each food parameter based on its effect on 6 key inflammatory biomarkers: IL-1β, IL-4, IL-6, IL-10, TNF-α, and CRP.^[15]^ Due to limitations in the NHANES data, this study included only 28 dietary components for the calculation of the DII.^[19]^ The components evaluated in this study include the following nutrients and food items: energy, protein, total fat, saturated fat, monounsaturated fat, polyunsaturated fat, cholesterol, carbohydrates, fiber, sugars, vitamin a, vitamin c, vitamine, folate, iron, magnesium, potassium, zinc, calcium, phosphorus, beta-carotene, lycopene, trans fats, omega-3 fatty acids, omega-6 fatty acids, flavonoids, tea (specific types of tea), spices (including some common spices known for anti-inflammatory properties). Each nutrient is associated with an inflammatory effect score, with positive values indicating pro-inflammatory effects and negative values indicating anti-inflammatory effects.^[20]^ The score for each nutrient changes according to its intake level, which is weighted and summed to produce the final DII score.^[21]^ Previous studies have demonstrated that the accuracy of DII calculation is not significantly affected when fewer than 30 dietary components are used.^[20]^

### 2.3. Assessment of overactive bladder (OAB)

The history of OAB was collected through a face-to-face interview conducted by trained staff using a standardized questionnaire for urge incontinence and nocturia. The presence and severity of urge incontinence were assessed using 2 questions: “During the past 12 months, have you/has SP leaked or lost control of even a small amount of urine due to an urge or pressure to urinate, and couldn’t get to the toilet fast enough?” and “How frequently does this occur?.” Nocturia was measured by asking, “During the past 30 days, how many times per night did you/SP most typically get up to urinate, from the time you/s/he went to bed until the time you/he/she got up in the morning?” The OAB Severity Score was calculated, with a total score of ≥3 used to diagnose OAB.^[22-24]^ These measures are consistent with previous studies utilizing the NHANES database.^[25]^ All the relevant questions and scores are given in Table 1.

**Table 1:** Criteria for conversion of symptom frequencies recorded in NHANES and OABSS scores.

According to NHANES score	According to OABSS score
Urge urinary incontinence frequency	Urge urinary incontinence score
Never	0
Less than once a month	1
A few times a month	1
A few times a week	2
Every day or night	3
Nocturia frequency	Nocturia score
0	0
1	1
2	2
3	3
4	3
5 or more	3
When total score ≥3, the diagnosis is OAB	

NHANES = National Health and Nutrition Examination Survey, OAB = overactive bladder, OABSS = overactive bladder syndrome score.

### 2.4. Assessment of covariates

Variables were collected from the NHANES database that could potentially impact the results of the analysis, including age, gender, race/ethnicity, education level, marital status, poverty–income ratio (PIR), hypertension, body mass index (BMI), diabetes, and sleep disorders. Age was categorized into 3 groups: 20 to 39, 40 to 59, and 60 and older. Race/ethnicity was categorized as: Mexican American, other Hispanic, non-Hispanic white, non-Hispanic black, and other race. Education level was categorized as: below high school, and high school and above. Marital status was categorized as: separated/widowed, married/living with partner, and never married. Household income, expressed as PIR, was categorized as: <1.3 (low), 1.3–3.5 (medium), and more than 3.5 (high). BMI was classified as: <25 (normal weight), 25 to 30 (overweight), and more than 30 (obese), following World Health Organization guidelines.

### 2.5. Treatment of missing values

Among the participants included in the study, some covariate data were missing, which could introduce bias in the results and affect the interpretation and credibility of the findings. To address this issue, we used multiple imputation to process and fill in the missing data.

### 2.6. . *Statistical analysis*

In our study, the variable ‘WTMEC2YR’ was used as a weight, and continuous variables are expressed as mean ± standard deviation. *P*-values for continuous variables were calculated using a weighted linear regression model. For categorical variables, percentages (weighted N, %) and *P*-values were determined using a weighted chi-square test. Three models were constructed for the study: crude model 1 was not adjusted for any covariates, model 2 was adjusted for age, gender, and race, and model 3 was adjusted for age, gender, race/ethnicity, education level, marital status, PIR, hypertension, BMI, diabetes, and sleep disorders. Multiple logistic regression models were used to examine the associations between DII and OAB. Potential nonlinear relationships between DII and OAB were further explored using smoothed curve fitting. *P*-values <.05 were considered statistically significant. All analyses were conducted using R software, version 4.2.1.

## 3. Results

### 3.1. Basic characteristics of the study population

A total of 21,263 participants were included in this study, of whom 25.2% had OAB and 74.8% were non-OAB, a higher percentage of OAB patients were female (62.6%), those over 60 had a higher prevalence of OAB (53.4%) than those in other age groups. Among different races, Non-Hispanic White had a higher prevalence of OAB (44.3%), weight was positively correlated with OAB prevalence, and PIR was negatively associated with OAB prevalence. Hypertension, diabetes mellitus, and sleep disorders were all associated with the development of OAB (*P* < .05; Table 2).

**Table 2:** Baseline characteristics of all participants were stratified by overactive bladder (OAB), weighted.

	Total	OAB		*P* value
	(n = 21263)	No (n = 15888)	Yes (n = 5375)	
Age (yr)				<.001
20–40	7242 (34.06)	6466 (40.69)	776 (14.44)	
40–60	7247 (34.08)	5521 (34.75)	1726 (32.11)	
>60	6774 (31.86)	3901 (24.56)	2873 (53.45)	
Gender (%)				<.001
Male	10,499 (49.38)	8489 (53.43)	2010 (37.39)	
Female	10,764 (50.62)	7399 (46.57)	3365 (62.61)	
Race/ethnicity (%)				<.001
Mexican American	2951 (13.87)	2249 (14.16)	702 (13.06)	
Other Hispanic	2065 (9.71)	1559 (9.81)	506 (9.44)	
Non-Hispanic White	9112 (42.86)	6731 (42.37)	2381 (44.29)	
Non-Hispanic Black	4484 (21.09)	3108 (19.56)	1376 (25.60)	
Other race	2651 (12.47)	2241 (14.10)	410 (7.61)	
Education (%)				<.001
Less than high school	4572 (21.51)	2996 (18.86)	1576 (29.32)	
High school and more than high school	16,691 (78.49)	12,892 (81.14)	3799 (70.68)	
Marital (%)				<.001
Widowed/divorced/separated	4799 (22.57)	2975 (18.73)	1824 (33.94)	
Married/living with partner	12,729 (59.86)	9843 (61.95)	2886 (53.69)	
Never married	3735 (17.57)	3070 (19.32)	665 (12.37)	
PIR (%)				<.001
0–1.5	7841 (36.88)	5515 (34.71)	2326 (43.27)	
1.5–3.5	6937 (32.62)	5094 (32.06)	1843 (34.28)	
>3.5	6485 (30.50)	5279 (33.23)	1206 (22.45)	
Hypertension (%)				<.001
Yes	7973 (37.49)	4933 (31.05)	3040 (56.56)	
No	13,290 (62.51)	10,955 (68.95)	2335 (43.44)	
BMI (%)				<.001
0–25	7054 (33.18)	5583 (35.14)	1471 (27.37)	
25–30	7229 (33.99)	5497 (34.60)	1732 (32.22)	
>30	6980 (32.83)	4808 (30.26)	2172 (40.41)	
Diabetes (%)				<.001
Yes	2934 (13.79)	1624 (10.22)	1310 (24.37)	
No	17,787 (83.65)	13,931 (87.68)	3856 (71.74)	
Borderline	542 (2.55)	333 (2.10)	209 (3.89)	
Sleep disorder (%)				
Yes	5842 (27.48)	3746 (23.58)	2096 (39.01)	<.001
No	15,421 (72.52)	12,142 (76.42)	3279 (60.99)	<.001
DII	1.48 ± 1.91	1.41 ± 1.90	1.71 ± 1.88	<.001

BMI = body mass index, DII = dietary inflammatory index, NHANES = National Health and Nutrition Examination Survey, OAB = overactive bladder syndrome, PIR = poverty–income ratio.

### 3.2. Relationship between DII and OAB

The correlation between DII and OAB was further validated using multiple logistic regression analysis. As shown in Table 3, when treated as a continuous variable, DII was positively correlated with the risk of OAB in model 3, which was fully adjusted for various covariates (OR 1.18, 95% CI 1.16 to 1.21), meaning that for every unit increase in DII, the risk of OAB increased by 18%. DII was also analyzed by quartiles, with the first quartile (Q1) used as the reference. In the unadjusted model, the risk of OAB was elevated by 41% in the highest quartile (Q4; OR 1.41, 95% CI 1.38–1.65). In model 2, which was adjusted for sex, age, and race/ethnicity, the risk of OAB was elevated by 21% at the Q4 level compared to Q1 (OR 1.21, 95% CI 1.10–1.34). Finally, in model 3, which adjusted for education level, marital status, PIR, hypertension, BMI, diabetes, and sleep disorders, the risk of OAB at the Q4 level was elevated by 20% (OR 1.20, 95% CI 1.01–1.42; *P* for trend <.001).

**Table 3:** Association between dietary inflammatory index (DII) and overactive bladder (OAB), NHANES 2011–2018.

Characteristic	Model 1^*^		Model 2^†^		Model 3^‡^	
	OR (95% CI)	*P* value	OR (95% CI)	*P* value	OR (95% CI)	*P* value
DII	1.09 (1.07, 1.11)	<.001	1.12 (1.10, 1.17)	<.001	1.18 (1.16, 1.21)	<.001
DII(categorical)						
Q1	Reference		Reference		Reference	
Q2	1.12 (1.03, 1.23)	.01	1.06 (0.97, 1.17)	.20	1.04 (1.02, 1.08)	<.001
Q3	1.28 (1.12, 1.40)	<.001	1.09 (0.99, 1.21)	.05	1.23 (1.08, 1.54)	<.001
Q4	1.41 (1.38, 1.65)	<.001	1.21 (1.10, 1.34)	.00	1.20 (1.01, 1.42)	<.001
*P* for trend		<.001		<.001		<.001

BMI = body mass index, DII = dietary inflammatory index, NHANES = National Health and Nutrition Examination Survey, OAB = overactive bladder syndrome, PIR = poverty–income ratio.

* Model 1, no covariates were adjusted.

† Model 2, age, gender, and race/ethnicity were adjusted.

‡ Model 3, age, gender, race/ethnicity, education level, marital status, PIR, hypertension, BMI, diabetes and sleep disorder were adjusted.

### 3.3. Nonlinear analysis

**Figure 2.**
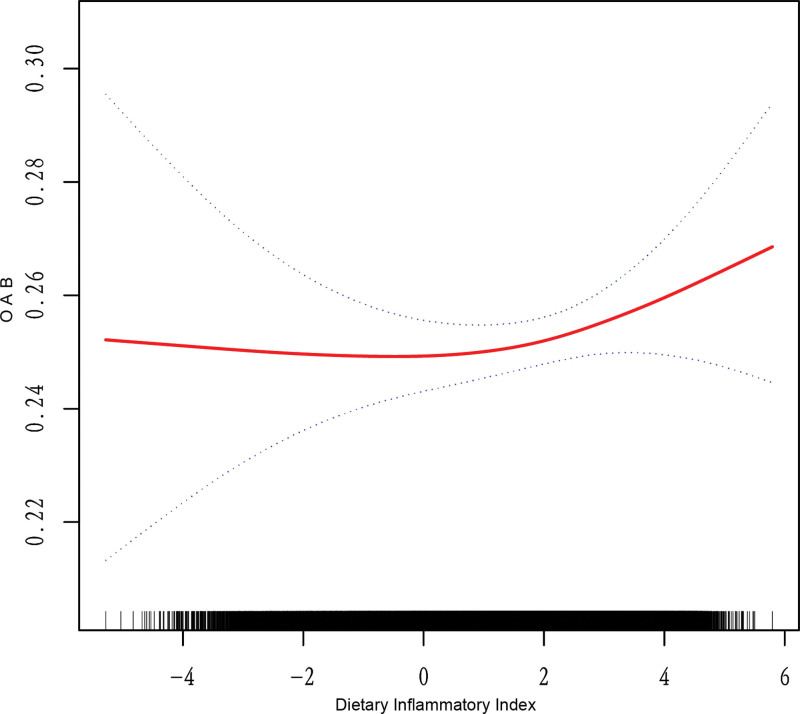
Nonlinear correlation between dietary inflammatory index (DII) and overactive bladder (OAB). DII = dietary inflammatory index, OAB = overactive bladder.

Smoothed curve fitting shows a U-shaped nonlinear relationship between DII and the risk of OAB. After adjusting for potential confounding variables such as age, gender, race/ethnicity, education level, marital status, PIR, hypertension, BMI, diabetes, and sleep disorders, the analysis reveals that when the DII score is below 1.92, changes in DII do not significantly affect OAB risk (OR 1.01, *P* = .92). However, above the threshold of 1.92, each unit increase in DII significantly increases the risk of OAB (OR 1.19, *P* < .001). The graph includes a logarithmic likelihood ratio test (*P* = .006), supporting the segmented model. When the level of DII was below the threshold of 1.92, changes in DII had no significant effect on the risk of OAB (OR 1.01, *P* = .92). The lack of significant effect below this threshold could be attributed to the possibility that low-to-moderate levels of dietary inflammation may not trigger the physiological changes required to alter bladder function (Fig. 2). Conversely, when the DII surpasses the inflection point, the body’s inflammatory response may become more substantial, potentially exacerbating bladder sensitivity and contributing to OAB risk. This aligns with existing literature, which suggests that low-to-moderate levels of inflammation may not manifest in clinical symptoms or conditions, whereas higher levels of inflammation are more likely to be pathophysiologically significant. However, when the level of DII exceeded this threshold, the risk of OAB increased significantly fwith every DII unit rise (OR 1.19, *P* < .001). The linear log-likelihood ratio test (*P* = .006) further supported that the segmented model was superior to the unilinear model, suggesting that elevated levels of DII may raise the risk of OAB (Table 4).

**Table 4:** Threshold effect analysis of the correlation between dietary inflammatory index (DII) and overactive bladder (OAB).

	Depression
Fitting by standard linear model	
OR (95% CI)	1.01 (0.99, 1.03)
* P*-value	<.001
Fitting by 2-piecewise linear model	
DII	
Breakpoint (K)	1.92
OR1 (<1.92)	1.01 (0.98, 1.02) 0.92
OR2 (> 1.92)	1.19 (1.05, 1.33) < 0.001
OR2/OR1	1.18 (1.12, 1.25) < 0.001
Logarithmic likelihood ratio test *P*-value	.006

DII = dietary inflammatory index, OAB = overactive bladder syndrome.

### 3.4. Subgroup analysis

**Figure 3.**
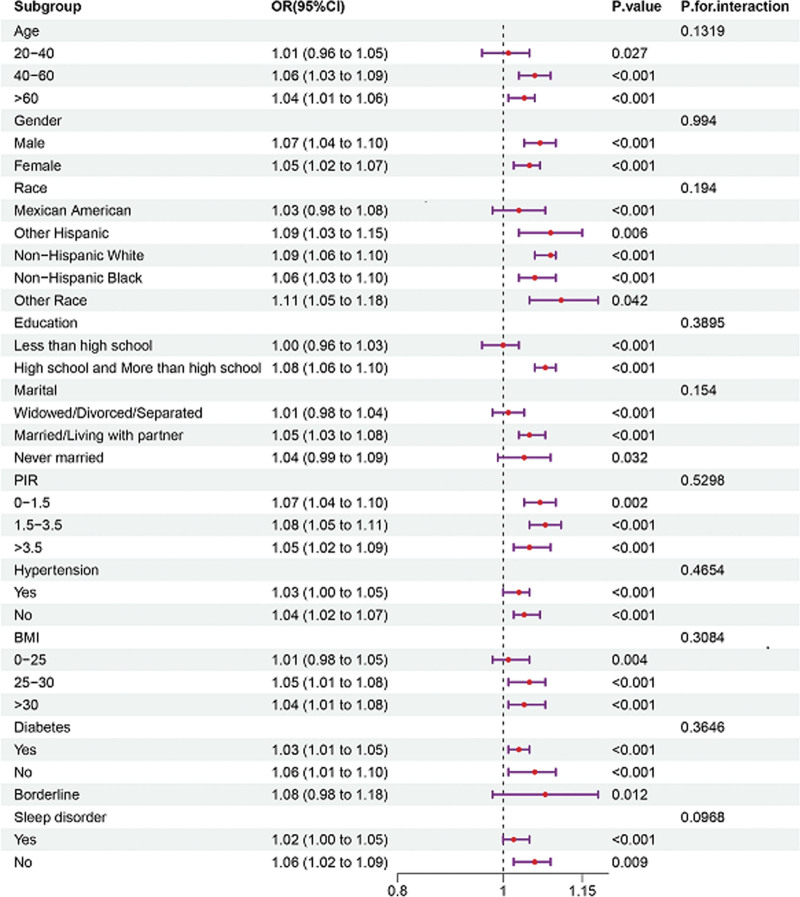
Subgroup analysis and interaction test between dietary inflammatory index (DII) and overactive bladder (OAB). BMI = body mass index, DII = dietary inflammatory index, NHANES = National Health and Nutrition Examination Survey, OAB = overactive bladder, PIR = poverty–income ratio.

This figure presents the results of subgroup analyses examining the relationship between DII and OAB risk across various demographic and clinical characteristics, including age, gender, race/ethnicity, education level, marital status, PIR, hypertension, BMI, diabetes, and sleep disorders. The analysis shows that there were no significant interactions (*P* for interaction > .05), suggesting that the relationship between DII and OAB remains consistent across different subgroups (Fig. 3).

### 3.5. Sensitivity analysis

To prevent the influence of missing values on the results, a sensitivity analysis was performed to exclude individuals with any missing variables. The sensitivity analysis showed that DII was positively correlated with the possibility of developing OAB, regardless of whether DII was treated as a continuous or categorical variable. This finding was consistent with the results of our study (Table 5).

**Table 5:** Relationship between DII and prevalence of OAB after excluding any missing values.

Characteristic	Model 1		Model 2		Model 3	
	OR (95% CI)	*P* value	OR (95% CI)	*P* value	OR (95% CI)	*P* value
DII	1.11 (1.07,1.17)	<.001	1.21 (1.19, 1.26)	<.001	1.19 (1.15, 1.22)	<.001
DII (categorical)						
Q1	Reference		Reference		Reference	
Q2	1.15 (1.12, 1.17)	.01	1.09 (1.01, 1.17)	.17	1.06 (0.98, 1.15)	<.001
Q3	1.36 (1.27, 1.40)	<.001	1.16 (1.07, 1.25)	0.02	1.24 (1.15, 1.34)	<.001
Q4	1.47 (1.37, 1.57)	<.001	1.28 (1.18, 1.38)	<.001	1.18 (1.15, 1.22)	<.001
*P* for trend		<.001		<.001		<.001

## 4. Discussion

This study comprised 21,263 people from the NHANES dataset, with 5375 diagnosed with OAB, representing approximately 25.2% of the population. Multivariate logistic regression analysis, adjusted for potential confounders, revealed that the risk of developing OAB at the highest level of DII (Q4) was 20.1% higher than at the lowest level (Q1). The results revealed that a higher DII was correlated with the possibility of developing OAB. Additionally, smoothed curve fitting demonstrated a U-shaped, nonlinear positive correlation.

between DII and OAB, with a point of inflection at 1.92. This correlation remained consistent across population subgroups in the stratified analysis.

Dietary conditions may be closely related to the development of OAB, acting as either a protective or risk factor.^[26,27]^ Several studies have demonstrated that the DII is significantly linked.

with a variety of diseases, including obesity,^[28]^ type 2 diabetes,^[29]^ metabolic syndrome,^[30]^ and coronary heart disease.^[31]^ Further research has highlighted the crucial role that both anti-inflammatory and pro-inflammatory dietary components play in the progression of these diseases, with changes in dietary composition leading to varying protective or detrimental effects.^[32]^ Current studies show that dietary components can either promote inflammation or have anti-inflammatory effects, depending on the specific nutrients involved. Some studies suggest that high consumption of fruits, vegetables, and other plant-based ingredients can effectively reduce oxidative stress.^[33]^ Additionally, intake of oleic acid, vitamin E, and similar nutrients has been linked to anti-inflammatory effects, helping to reduce inflammatory responses. Conversely, excessive intake of caffeine, cholesterol, and alcohol can induce diabetic neuropathy and atherosclerosis, triggering systemic inflammation and urinary epithelial dysfunction.^[34]^ An increased intake of these components can elevate DII, providing further insight into the relationship between DII and OAB. Moreover, diet can influence gut microbiome metabolism. High-fat diets, for instance, can alter gut microflora, increasing intestinal permeability and promoting inflammation in the body. Furthermore, a high-fat and high-cholesterol diet can lead to obesity, which is a major contributing factor to the onset of OAB, aligning with the findings of our opinions.

In the last few years, various research have investigated the pathogenesis of OAB, but the exact mechanisms remain unclear. However, several major areas of consensus have emerged. First, patients with OAB often exhibit increased sensitivity of the bladder mucosa, which is associated with bladder sensory neuropathy.^[35]^ One of the most common causes of peripheral neuropathy is diabetes mellitus, and the relationship between diabetes and OAB has been well-established.^[36]^ Moreover, carbohydrate intake can lead to fluctuations in blood glucose levels, suggesting that a higher carbohydrate intake may elevate DII scores, potentially increasing the risk of OAB. Second, individuals consuming high-fat and high-cholesterol diets are more prone to obesity, which in turn increases DII. Obesity results in an increase in abdominal and pelvic fat, which raises intravesical pressure, reduces bladder capacity, and predisposes individuals to OAB.^[37,38]^ Furthermore, oxidative stress and inflammation are pivotal factors in the etiology of OAB.^[39]^ These factors are closely linked to changes in the body’s inflammatory state, which can be triggered by various elements, including diet, nutritional status, and lifestyle habits. Dietary composition is particularly important, as anti-inflammatory components such as multivitamins and dietary fiber can effectively reduce oxidative stress and lower the risk of OAB. In contrast, pro-inflammatory components such as high-protein diets, excessive fatty acids, fat, and cholesterol intake may exacerbate inflammation, thereby increasing the risk of OAB. These findings provide a theoretical foundation for studying the connection between DII and OAB risk.

The results of these studies suggest that anti-inflammatory diets can effectively reduce the incidence of OAB, while pro-inflammatory diets increase the likelihood of OAB development. Moreover, consuming more anti-inflammatory foods reduces the DII, whereas a higher intake of pro-inflammatory components raises DII, consequently raising the likelihood of developing OAB. These outcomes support the conclusions drawn from our analysis, which found that the likelihood of OAB is positively correlated with the level of DII when the DII exceeds 1.92. In conclusion, these findings highlight that controlling DII within a specific range could become an essential strategy for preventing OAB.

## 5. Strengths and limitations

A major strength of this study is its large sample size, which enhances the statistical power and reliability of the findings. The link between the DII and the risk of OAB was clearly presented, showing that individuals with higher DII levels tend to have an elevated risk of OAB. Additionally, the credibility of our results was enhanced by subgroup and sensitivity analyses. This study suggests that modifying dietary composition may help reduce the risk of OAB.

However, several limitations should be acknowledged. First, our data were collected from the NHANES database, and the dietary intake information may be subject to recall bias, which could compromise the DII results’ accuracy. Furthermore, the cross-sectional design restricts the capacity to infer causal relationships between OAB risk and DII. To address these limitations, future research involving prospective cohort studies across diverse ethnic populations is warranted.

## 6. Conclusion

In conclusion, this study identifies a significant association between the DII and the risk of OAB in a large sample of US adults from the NHANES dataset. These findings suggest that dietary inflammation may play a role in the development of OAB, and that controlling DII through dietary interventions may hold potential as a preventive strategy for this condition. However, it is important to note that our study’s cross-sectional design limits our ability to infer causal relationships. While associations were observed between DII and OAB, cross-sectional data cannot establish temporality, meaning we cannot determine whether increased DII contributes to the onset of OAB or whether existing OAB influences dietary patterns. Therefore, causal inference can only be supported by longitudinal or interventional studies, which are necessary to further investigate the directionality and underlying mechanisms of this relationship.

## Acknowledgments

We would like to express our sincere gratitude to the National Center for Health Statistics (NCHS) for providing access to the National Health and Nutrition Examination Survey (NHANES) data used in this study.

## Author contributions

**Conceptualization:** Mingchu Jin, Maohua Luo.

**Data curation:** Mingchu Jin, Yuzhu Huang.

**Investigation:** Maohua Luo.

**Methodology:** Mingchu Jin, Haidong Hao, Maohua Luo.

**Project administration:** Mingchu Jin.

**Resources:** Mingchu Jin, Yuzhu Huang, Xintao Lv, Haidong Hao.

**Software:** Mingchu Jin, Xintao Lv.

**Validation:** Mingchu Jin, Haidong Hao, Maohua Luo.

**Writing – original draft:** Mingchu Jin, Xintao Lv.

**Writing – review & editing:** Mingchu Jin, Maohua Luo.
